# Using the tools of genetic epidemiology to understand sex differences in neuropsychiatric disorders

**DOI:** 10.1111/gbb.12660

**Published:** 2020-06-22

**Authors:** Alison K. Merikangas, Laura Almasy

**Affiliations:** ^1^ Department of Biomedical and Health Informatics Children's Hospital of Philadelphia Philadelphia Pennsylvania USA; ^2^ Penn‐CHOP Lifespan Brain Institute University of Pennsylvania Philadelphia Pennsylvania USA; ^3^ Department of Genetics, Perelman School of Medicine University of Pennsylvania Philadelphia Pennsylvania USA

**Keywords:** family study, genetic epidemiology, multifactorial polygenic model, psychiatric disorders, sex differences, substance use disorder, twin study

## Abstract

Many neuropsychiatric disorders exhibit differences in prevalence, age of onset, symptoms or course of illness between males and females. For the most part, the origins of these differences are not well understood. In this article, we provide an overview of sex differences in psychiatric disorders including autism spectrum disorder (ASD), attention deficit/hyperactivity disorder (ADHD), anxiety, depression, alcohol and substance abuse, schizophrenia, eating disorders and risk of suicide. We discuss both genetic and nongenetic mechanisms that have been hypothesized to underlie these differences, including ascertainment bias, environmental stressors, X‐ or Y‐linked risk loci, and differential liability thresholds in males and females. We then review the use of twin, family and genome‐wide association approaches to study potential genetic mechanisms of sex differences and the extent to which these designs have been employed in studies of psychiatric disorders. We describe the utility of genetic epidemiologic study designs, including classical twin and family studies, large‐scale studies of population registries, derived recurrence risks, and molecular genetic analyses of genome‐wide variation that may enhance our understanding sex differences in neuropsychiatric disorders.

## Graphical abstract

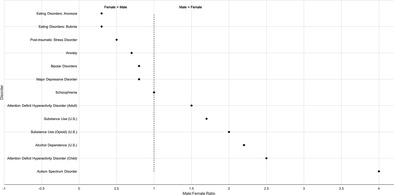

## 1 INTRODUCTION

The National Institutes of Health (NIH) mandate to consider sex differences in both human and basic research (ie, “that scientists will account for the possible role of sex as a biological variable in vertebrate animal and human studies” (notice no. NOT‐OD‐15‐102) has led to renewed interest in studying potential hypotheses for sex differences in traits and diseases. Even though human studies of mental disorders have generally considered sex differences in etiologic and treatment studies, few basic studies of their underlying biology have investigated sex differences. The NIH mandate for inclusion of sex as a biological variable will help to align the findings from human and animal studies^1^ and ultimately will assist in determining the etiology and treatment of mental disorders.

Sex differences in a disease or trait can provide insight into its causes, risk factors, and consequences. The aims of this paper are to: (a) summarize the sex‐specific lifetime prevalence of the most common psychiatric disorders among adults and youth; (b) enumerate hypotheses for sex differences in mental disorders; (c) describe the use of the concepts and tools of genetic epidemiology to evaluate sex differences in psychiatric disorders and (d) examine how traditional family and twin studies, and case‐control genome‐wide association studies (GWAS) can help to elucidate the etiology of psychiatric disorders in the molecular era. In examining the sex‐specific presentation of psychiatric disorders, we will consider sex differences in lifetime prevalence, onset, severity and/or clinical manifestations of these conditions. The hypotheses put forth to explain sex differences in mental disorders include artifactual or methodological differences in the studies or samples, differential expression or severity of disorders in males and females, sex differences in developmental trajectories, environmental factors, and different genetic architecture of the condition in males and females. As described below, the tools of genetic epidemiology, including family and twin study designs, can be used to evaluate potential explanations for sex differences, and may provide insight into the roles of both genetic and environmental factors in disease etiology.

## 2 SEX DIFFERENCES

**FIGURE 1:**
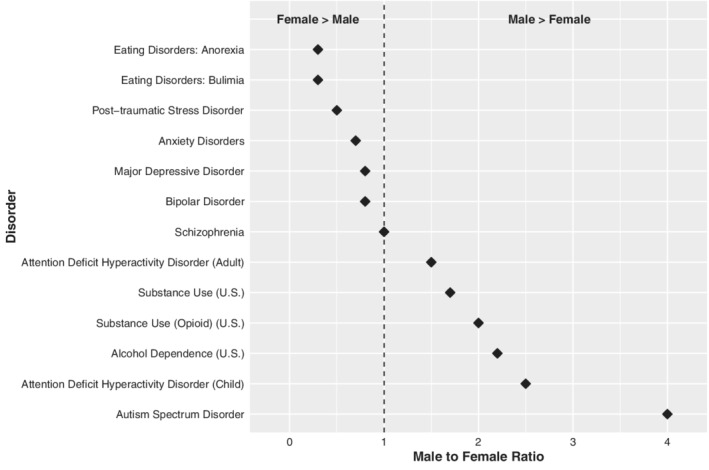
Male to female sex ratio of psychiatric disorders, sorted from most to least prevalent among females

Sex differences in the prevalence of mental disorders have long been established.^2^, ^3^, ^4^, ^5^ Irrespective of the absolute rates of disorders, the sex ratio for specific classes of mental disorders is quite consistent in community surveys of both youth and adults. Table 1 presents sex differences in lifetime prevalence, onset, and severity or clinical manifestations of psychiatric disorders. As described in Table 1, there is a female preponderance of mood disorders (male:female ratio [M:F] 0.8), anxiety disorders (M:F 0.7) and eating disorders (M:F 0.3), and males have greater rates of alcohol (M:F 2.2) and substance use (M:F 1.7) disorders, and behavior disorders (ie, attention deficit hyperactivity disorder [ADHD], oppositional defiant disorder [ODD] and conduct disorder [CD]).^6^, ^7^ Not all disorders exhibit these disparities in prevalence between males and females. The sex ratio is approximately equal for bipolar disorder and for schizophrenia.^8^ Studies of childhood conditions show that the rates of neurodevelopmental disorders (eg, autism spectrum disorder (ASD, M:F 4.0), ADHD (M:F 1.5) and CD) are substantially greater in males than females. Rates of some of the consequences of psychiatric disorders such as completed suicide are also higher among men than women (M:F 3.9).^9^ Figure 1 displays the sex ratios for all of the disorders listed in Table 1.

**TABLE 1:** Sex differences by disorder

Disorder	Male (%)	Female (%)	M:F ratio	Peak onset (years)	Severity/manifestations	Reference
Attention Deficit Hyperactivity Disorder (adult)	3.4	2.2	1.5	<18	Hyperactivity and impulsivity more common in males, while inattention is more common in females.	147
Attention Deficit Hyperactivity Disorder (child)	10.0	4.0	2.5	<5		148
Alcohol Dependence (U.S.)	17.4	8.0	2.2	25‐30	Males report drinking more frequently and in greater quantities, have higher rates of heavy episodic drinking (5+ drinks per occasion) and adverse drinking consequences.	7
Anxiety Disorders	22.2	33.3	0.7	12‐18	Greater symptom severity and more comorbidity in women than men.	149
Autism Spectrum Disorder	0.8	0.2	4.0	<6	Females tend to have less severe ASD and greater symptom improvement across development than males.^11^	150
Bipolar Disorder	0.6	0.8	0.8	25‐29	Severity and course are similar across sexes.	151
Eating Disorders: Anorexia	0.3	0.9	0.3	15‐20	No differences in severity.	152
Eating Disorders: Bulimia	0.5	1.5	0.3	20‐30	Greater severity for females.	152
Major Depressive Disorder	11.7	14.4	0.8	25‐40	No sex differences in remission, recurrence or chronic course. Women report more atypical symptoms, including increased appetite and hypersomnia.	153
Posttraumatic Stress Disorder	4.1	8	0.5	NA	Age at onset and course are similar, although women show greater symptom severity. The prevalence among women decreases with age.	39
Schizophrenia	0.5	0.5	1.0	18‐25	Men have a history of more pre‐ and peri‐natal complications, earlier age at onset, worse course and poorer response to typical antipsychotic medications than do women.^3^	154, 155
Substance use (U.S.)	3.3	2.0	1.7	20‐25	Adverse medical, psychiatric and functional consequences associated with SUDs are often more severe in women. No difference in treatment outcomes.	7
Substance use (Opioid) (U.S.)	0.10	0.05	2.0	25‐35		156

### 2.1 Sex differences in developmental trajectories

The sex ratio for many psychiatric disorders changes across development. In childhood, males have higher rates of neurologic and neurodevelopmental disorders including ASD, ADHD and learning disabilities, with an average 3:1 sex ratio for these conditions.^10^ Although males have higher rates of ASD than females, females may actually have greater remission or recovery from early childhood symptoms across development.^11^ Rates of behavioral, or externalizing disorders, such as ADHD, ODD and CD, are higher in males in childhood, and the male preponderance continues into adulthood, with a steeper increase in prevalence with increasing age.^12^ Similarly, during adolescence males and females initiate substance use at comparable rates, but males increase use faster than females.^13^ By contrast, prospective community studies (eg, The Great Smoky Mountains Study^14^) have shown that the prevalence of mood and anxiety disorders (ie, internalizing disorders) tends to be similar in boys and girls prior to adolescence, but the sex ratio diverges at adolescence with females having higher rates throughout adulthood.^15^ Bulimic symptoms also differ between boys and girls across development. Prospective research that shows an increase in symptoms in girls between ages 14 and 16, but a decrease among boys across the same period. The severity of bulimic symptoms is greater for girls across all ages.^16^ Symptoms of ADHD also change throughout development, especially for males, who tend to exhibit more hyperactivity and impulsivity in childhood. During adolescence, the level of hyperactivity‐impulsivity in boys declines to the same level as girls, whereas inattentive symptoms are similar in males and females and steady across development.^17^ Sex differences in onset and course of disorders of mid and late life have been extensively documented for several neurologic conditions, such as migraine and Alzheimer's disease, in which both the risk factors and manifestations are divergent in males and females.^18^ For mental disorders, the course of schizophrenia tends to be less severe in women than in men,^3^ and males appear to have more negative consequences of mood disorders, particularly bipolar disorder, including suicide, substance use disorders and cognitive decline.^19^

### 2.2 Differential manifestations by sex

Sex differences in symptom presentation may influence the sex ratio in neurodevelopmental disorders, such as ADHD and ASD. Among cases of ADHD, approximately 80% of child clinic cases are male, whereas this decreases to approximately 50% in adult clinics. It has been hypothesized that boys show higher levels of hyperactivity and impulsivity, while girls manifest more inattentive symptoms and tend to be less disruptive,^20^ and therefore less likely to receive a diagnosis. Similarly, it has been suggested that the sex ratio in ASD is the result of underdiagnosis in girls, because they tend to develop and maintain more language and cognitive skills than do boys. In addition, boys show higher levels of restricted/repetitive behaviors and interests than females, whereas there appear to be no gender differences in social interaction or communication overall.^21^ Although the prevalence of schizophrenia is approximately equal in males and females, males with this disorder also have been shown to suffer from more severe symptoms, course and impact than females.^22^

Similar to increasing attention to cardiovascular^23^ and other physical conditions,^24^ sex differences in the expression of mental disorders have been widely studied. Differential expression could be attributable to neurobiological factors, such as the fluctuation of reproductive hormones or immune system regulation, greater exposure or sensitivity to environmental stressors or exposures or genetic factors that could play a role in sex specific expression independently or in combination, as discussed below.

In summary, almost all common psychiatric conditions show differences by sex in prevalence, course and/or severity. In general, males show higher prevalence and greater severity of neurodevelopmental disorders, behavior disorders and substance use disorders whereas females exhibit higher prevalence and greater severity of mood disorders, eating disorders and anxiety disorders.

## 3 POTENTIAL EXPLANATIONS FOR SEX DIFFERENCES

### 3.1 Artifactual or methodologic

The unequal sex ratio for several of the classes of mental disorders could be due to various methodological factors including ascertainment biases, differential reporting or recognition by males and females or factors associated with assessments that preferentially identify symptoms/disorders by sex. Ascertainment biases in clinical samples in psychiatry have been well‐recognized,^25^ and the large‐scale community surveys of both adults and children have showed biases in severity and comorbidity of clinical samples, and under‐or‐over‐representation by sex. Artifactual differences could also be due to misclassification based on symptom presentation or severity, and social or cultural differences in the recognition and interpretation of symptoms. For example, the higher rate of hyperactivity symptoms in males noted above may contribute to the higher rate of ADHD diagnoses in males. A longitudinal cohort study that tracks sex‐specific incidence throughout the period of risk in a community based or high‐risk sample would be one approach to test whether the deviant sex ratio for a particular disorder is a result of sampling or diagnosis. For example, depressive symptoms present equally in boys and girls in childhood, and the nearly 2‐fold increase among depressive symptoms among women only appears postpubertally.^26^ Therefore, sex differences in the course or trajectory of depression that have been shown in longitudinal cohort studies^27^, ^28^ may not be evident in a cross‐sectional examination.

In general, females are more likely to report symptoms and to utilize medical services.^24^ Sex differences in the recognition or reporting of symptoms of a disorder have been widely studied for mental disorders, particularly depression. Women have been shown to be more aware of psychological symptoms, and to report those that are present.^29^ Sociocultural factors may also lead to reduction of reporting of symptoms in males. For example, some depressed men may attempt to hide their emotions and appear to be angry or aggressive instead of sad, which may lead to lack of recognition of depressed mood.^30^

### 3.2 Environmental factors

The most obvious hypothesis for the increased risk of mood and anxiety disorders in females is the influence of sex hormones (specifically, estradiol and progesterone) across the life span. In fact, the increased female: male ratio in depression at adolescence is directly proportional to development of female reproductive function, that is a marked increase at the onset of puberty.^31^

Prenatal environmental exposures may influence sex differences during the intrauterine period, at delivery or throughout the life span of the individual. Infections, dietary factors, drug exposures or perinatal complications could differentially influence brain development in male or female offspring (eg, McCarthy et al.^32^). Studies of rodents have shown widespread sex differences in brain structures according to the timing and dose of exposure to sex steroids, and the production of testosterone in male fetuses has been shown to induce sexual differentiation in the brain. Specifically, rodent studies have showed that even though female fetuses do not produce testosterone, they do respond to exogenous testosterone in the prenatal environment (ie, from male littermates), and remain sensitive to its masculinizing effects for a longer time than males, even postnatally.^33^ For twin pregnancies, sex of the co‐twin has even been proposed to influence manifestations of neurodevelopmental disorders through effects on the intrauterine environment. For example, Eriksson and colleagues^34^ investigated whether elevated levels of testosterone in utero increase the risk of developing ASD or ADHD traits, by assuming fetuses with a male co‐twin will be exposed to higher levels of endogenous testosterone than fetuses with a female co‐twin, leading to greater masculinization of the brain. Their data did not support this hypothesis, and instead they reported that presence of a female co‐twin corresponded to a greater risk for ASD or ADHD traits.^34^ Recent advances in neuroscience suggest that the female brain may exhibit greater plasticity in response to challenges.^35^ This would be expected to lead to lower prevalence of cognitive dysfunction or other disorders, such as ASD, which may be influenced by such environmental exposures. In fact, one study demonstrated testosterone levels in boys mediated prefrontal‐hippocampal covariance, but this was not shown in girls.^36^

Differential exposure or reactivity to postnatal environmental factors between males and females could also contribute to sex differences in mental disorders across the lifespan. With respect to other body systems, data suggest that females may suffer from more detrimental effects of smoking (eg, developing chronic obstructive pulmonary disease, COPD),^23^ and alcohol misuse^37^ than do males. More recently, sex differences in the microbiome have been proposed to protect males against autoimmune disorders.^38^ Differential susceptibility to environmental exposures across the lifetime should also be considered in examining causes of sex differences in psychiatric disorders. For example, women are twice as likely as men to experience posttraumatic stress disorder (PTSD), even though men and women are exposed to traumatic events at approximately equal rates. However, the type of traumatic event varies by sex; women are more likely to experience intimate partner violence, sexual assault and childhood maltreatment, while men are more likely to have experienced accidental injury, nonsexual physical assault and war‐related events.^39^

Differences in exposure or reactivity to environmental factors have also been widely studied as an explanation for increased rates of mood disorders among females. Women have tended to experience higher rates of sexual abuse as children and interpersonal violence as adults, as well as other interpersonal stressors, including societal gender inequality and discrimination.^40^ Women may also be more predisposed to mood disorders due to increased psychological sensitivity and lower self‐esteem than men.^40^ In contrast, men have been shown to experience a need to conform to specific masculine gender roles that may inhibit their reporting of depressive or anxiety symptoms due to implications for perceived weakness, or strength.^41^ This under‐reporting may also reduce treatment‐seeking behavior in men.^41^ There is also evidence that similar life events may have differential influences on mood disorders in males and females. Using the opposite sex twin approach (discussed below), Kendler and colleagues^42^ found that whereas acute stressors and prior depression and behavioral disorders were associated with depression in males, interpersonal relationships combined with temperamental factors had greater influence in the onset of depression in females.

### 3.3 Genetic factors

The role of genetic factors in sex differences in a trait or disorder will differ according to the genetic architecture of the condition. Based on our current understanding, we anticipate that most common psychiatric disorders are polygenic and reflect the combined contributions of hundreds or thousands of genes^43^ with a small subset of individuals having rare variants of larger effect, such as Fragile X in ASD or chromosome 22q11 deletion in schizophrenia. Genetic factors may contribute to sex differences either through the systematic differences between males and females in sex chromosome composition or through genotype‐by‐sex interactions resulting in differential impact of identical autosomal genetic variants in males and females.

### 3.4 Sex chromosomes

An obvious genetic hypothesis for sex differences in a heritable trait or disorder is that it is a manifestation of genes on the sex chromosomes. X‐linked inheritance is one of the most important sources of sex differences in disease. In fact, early work in the familial transmission of bipolar disorder were consistent with X‐linkage,^44^ that is, a lack of male‐to‐male transmission, and an increased risk of disease in female relatives.^45^

Sex chromosome aneuploidies (SCA) may provide insight into the sex‐chromosome impact on sex differences in psychiatric disorders. In particular, if risk of a disorder tracks with the dosage of X or Y chromosomes, this could suggest a mechanism for observed male‐female differences. As described by Green and colleagues,^46^, ^47^ sex chromosome number has been associated with a range of behavioral phenotypes, and can provide clues to sex chromosome effects on neurodevelopment. Printzlau and colleagues^48^ describe the cognitive, behavioral and neural correlates of sex chromosome aneuploidies. On average, people with sex chromosome aneuploidies have greater prevalence rates of ASD and ADHD, as well as cognitive deficits as measured by full‐scale, verbal and performance IQ.^48^ However, brain volume differences are more variable, with some evidence that dosage of the X chromosome is related to reduced brain volume (eg, greater reduction in XXY, XXX and XXYY carriers compared with XY and XX, but no brain volume differences in XO and XYY carriers).^48^

X‐inactivation (lyonization), the compensatory mechanism by which balanced gene dosage is achieved between (XY) males and (XX) females is another important concept in investigating sex differences in disease. In general, there is random inactivation of one X chromosome that varies in each cell. However, one intriguing finding that is worthy of further study is the extent to which some genes escape X‐inactivation.^49^ Approximately 15% of genes on the X chromosome are not inactivated in females. Among males no differences in expression levels between escape genes and inactivated genes have been reported; however, the degree to which escape genes are inactivated in females varies between cells, tissues, genes and individuals.^50^ Moreover, it is theorized that genes outside of the pseudo‐autosomal region must be upregulated on the male X in order to maintain function.^48^ X‐inactivation escape genes have been associated with cognitive impairment,^51^ and a recent report shows an association between genes that escape X inactivation and sex differences in the prevalence of comorbid musculoskeletal pain and posttraumatic stress symptoms after motor vehicle accidents.^52^ There has been little study of the role of X inactivation in psychiatric disorders, but given the established association of inactivation with cognitive impairment and the known role of cognitive function as an endophenotype for some psychiatric disorders, this is a mechanism that deserves further consideration.

One widely studied specific sex chromosome alteration that has been associated with neuropsychiatric conditions is Fragile X syndrome (FXS), which is caused by the expansion of a trinucleotide repeat in the Fragile X Mental Retardation 1 (*FMR1*) gene on the X chromosome. In addition to being the most common inherited cause for ASD and intellectual disability, deletion of the *FMR1* gene is also associated with a broad range of neuropsychiatric outcomes in both youth and adults ranging from anxiety disorders to substance abuse.^53^ Studies of female carriers of the Fragile X mutation have also informed sex differences in the influence of these mutations.^54^ Premutation carriers of the *FMR1* gene have a lesser number of repeats (55‐200 CGG repeats) than those who manifest FXS (>200 CGG repeats), and increased prevalence of anxiety, depression, ASD, ADHD, intellectual and learning disabilities, substance use problems, and personality disorders have been reported.^53^, ^55^ In addition, a number of X chromosome genes and copy number variants (CNV) have been associated with intellectual disability,^56^ developmental delay,^57^ and schizophrenia,^58^ but the risk of incurring a mutation associated with intellectual disability on the X chromosome is the same for males and females.

Another severe X‐linked neurodevelopmental disorder is Rett syndrome (RTT), that is caused by mutations in the transcriptional regulator *MECP2*, an X chromosome gene. Similar to FXS, symptoms typically include language delays, motor coordination problems and repetitive movements.^59^ Originally Rett was thought to be fatal in males, and only manifested in females. However, like other X chromosome genes, *MECP2* is subject to X‐inactivation, and most affected individuals are female heterozygotes who display cellular mosaicism for normal and mutant *MECP2*. Males rarely survive, but those who do, and are hemizygous for mutant *MECP2* are more severely affected than heterozygous females.^60^

### 3.5 Multifactorial polygenic model

With growing evidence for the polygenicity of mental disorders,^61^ sex differences in the prevalence of disorders could also reflect differences in thresholds for manifestation of mental disorders based on differential accumulation of risk factors for these conditions, as proposed by the multifactorial polygenic threshold model (see Falconer, described below).^62^

In summary, sex differences in the rate and presentation of psychiatric disorders reported in studies to date may be due to an artifact of reporting, sampling bias, or true male‐female differences in their incidence or prevalence. Sex differences may also be the result of differential impact of pre‐ or post‐natal environmental exposures, genetic effects arising from sex chromosome composition, or differing polygenic liability thresholds in males and females.

## 4 APPLICATIONS OF GENETIC EPIDEMIOLOGY METHODS TO STUDY POTENTIAL EXPLANATIONS FOR SEX DIFFERENCES

The sub‐discipline of genetic epidemiology focuses on identification of the role of genetic factors and their joint influence with environmental factors in disease etiology. Genetic epidemiology employs traditional epidemiologic study designs including case‐control and cohort studies to evaluate the aggregation of disorders in groups as closely related as twins or as loosely related as migrant cohorts. Prior to the molecular genetic era, study designs in genetic epidemiology were designed to infer genetic causation by controlling for genetic background while letting the environment vary (eg, half‐siblings, separated twins) or conversely, controlling for the environment while allowing variance in the genetic background (eg, siblings, twins, adoptees, nonbiologic siblings).^63^ Investigations in genetic epidemiology are typically based on a combination of study designs including family, twin and adoption studies. As described below, sophisticated methods have recently been developed to compare combinations of genetic markers between cases and controls (eg, polygenic risk scores, PRS), and to conduct genome‐wide complex trait analysis (GCTA), that estimates the proportion of phenotypic variance explained by genetic variants (typically single nucleotide polymorphisms, SNPs) for complex traits.^64^ Some additional genetic epidemiology terminology is included in Box 1.

BOX 1 **Genetic epidemiology terminology**

*Heritability*: The extent to which variation in a trait is due to variation in genetic factors.

*Broad‐sense heritability*: *H* ^2^ = *V*_G_/*V*_P_, the proportion of the overall phenotypic variation (*V*_P_) due to genetic values (*V*_G_) that may include additive, dominance and epistasis effects.

*Narrow‐sense heritability*, *h* ^2^ = *V*_A_/*V*_P_, the proportion of phenotypic variation that is due to additive genetic values (*V*_A_).

*Familial relative risk*: Disease risk in relatives of cases vs controls.

*Genetic attributable risk*: The proportion of risk for a disease that would be eliminated if a particular gene or genes were not involved in the disease.

*Threshold liability model*: Disease model proposed by Falconer (1965) that posits a continuous underlying liability to a disease, ranging from 0 to 1, based on additive cumulation of many genetic and environmental risk factors, with a threshold that defines the point after which the disease is manifest.

*Sex‐dependent liability model*: Disease model proposed by Carter (1969), wherein one sex requires a greater genetic liability to manifest a disease.

**FIGURE 2:**
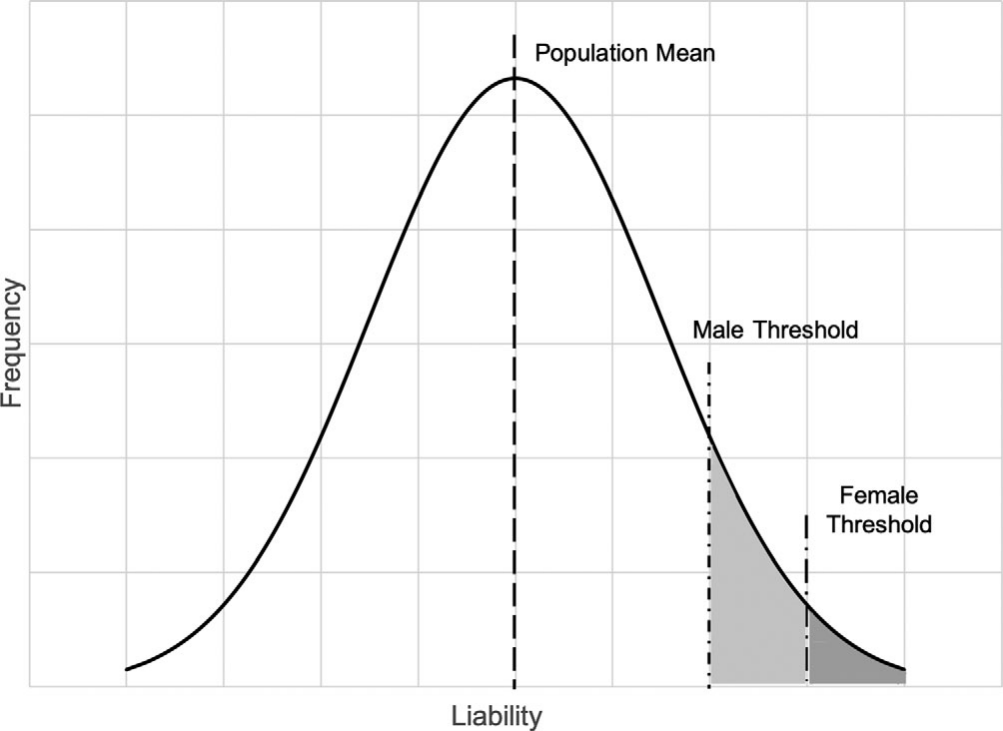
The general multifactorial model of disease transmission.^65^ The liability, or propensity for transmitting the disorder, is plotted on the *X*‐axis. The frequency distribution in the population is plotted on the *Y*‐axis. The shaded sections represent the proportion of affected individuals, light gray for males and dark gray for females. The multifactorial model specifies that numerous genetic and environmental factors is involved in an individual's liability for a particular disorder. The population mean (dash line) and threshold of liability after which the disorder becomes manifest is marked for males (dash dot line) and for females (long dash dot line). Sex differences in the liability to a condition may be tested by examining the risk to relatives of male versus female probands

Genetic epidemiologic study designs can be used to examine the extent to which genetic factors may explain sex differences in a trait or disease. In light of the evidence that most psychiatric disorders are multifactorial and polygenic, investigation of sex differences in transmission can be accomplished through application of the general multifactorial model of disease transmission, shown in Figure 2.^65^ The multifactorial model specifies that numerous genetic and environmental factors are involved in an individual's liability for a particular disorder. The liability, conceptualized as a continuous function that is assumed to be the result of an accumulation of multiple factors, including genetic risk loci, is plotted on the *X*‐axis. A threshold, or point at which the disorder becomes manifest based on population prevalence, is placed on the distribution to create a dichotomous distinction between affected and unaffected individuals. Therefore, unaffected individuals with lower aggregate risk fall below the threshold and affected individuals with higher underlying liability fall above the threshold. The shaded sections represent the proportion of affected individuals. The frequency distribution in the population is plotted on the *Y*‐axis.

This model was modified to test sex differences in the liability to a condition by applying separate thresholds for males and females by Carter,^66^ depicted in Figure 2 by light gray for males and dark gray for females.^66^ To test whether there is a sex difference in the transmission of a disorder, it is expected that the less frequently affected sex will have a higher threshold of liability for the disorder (ie, they require a greater number of genetic and/or environmental risk factors before manifesting the disorder than the more frequently affected sex). This implies that there is greater loading of risk factors among relatives of the less commonly affected sex, so their relatives would be more likely to manifest the disorder. This expectation can be tested systematically in family and twin studies through analysis of sex of proband effects as described below.^61^

### 4.1 Family studies

Familial aggregation is generally the first source of evidence that genetic factors may play a role in a disorder. The patterns of genetic factors underlying a disorder can be inferred from the extent to which patterns of familial resemblance adhere to the expectations of Mendelian laws of inheritance. Familial patterns may also be used to estimate the heritability of a trait or disorder, defined as the extent to which variation in a phenotype is attributable to genetic factors (see Box 1). Family studies can also be used to examine the extent to which familial/genetic factors may underlie sex differences in transmission or manifestation of a particular disorder.^67^ In the case of a female‐preponderant disorder, relatives of affected males would be expected to show higher rates of the disorder than do relatives of affected females. If the rates do not differ between the relatives of males and females with the disorder, then we can conclude that the sex difference in a particular condition cannot be attributed to familial or genetic factors.

Family studies of depression have yielded an average of a two to four times greater risk of depression in relatives of probands with depression than in controls.^68^ Several of the earlier family studies of depression that tested sex‐specific effects did not find evidence for a sex of proband effect in either adults,^69^, ^70^, ^71^ or youth, with equal rates of depression in relatives of male and female probands.^72^ A later summary of controlled family studies of depression did not find consistent evidence for sex differences in the transmission of depression in families.^68^ Likewise, family studies of bipolar disorder have not yielded evidence for sex differences in the familial transmission of this condition.^73^ Most of the more recent large scale family studies based on registry data tend to adjust for sex rather than systematically investigating its role in disease transmission.^74^, ^75^, ^76^

Findings on the role of sex differences in the transmission of schizophrenia have been inconclusive, due to differences in sampling, phenotypic definitions and study designs.^3^, ^77^, ^78^ However, Goldstein and colleagues^3^ propose that there may be sex differences in liability thresholds to schizophrenia due to greater impact of environmental exposures such as pre‐ and peri‐natal complications in boys. They observe varying rates of diagnoses across the schizophrenia spectrum in relatives of male and female probands, with higher rates of schizophrenia and schizoaffective disorder in families of female probands, and higher rates of schizotypal personality disorder in families of male probands. Therefore, this may indicate that males and females may exhibit different forms of the schizophrenia spectrum. This suggests that the systems underlying the illness may be different in males and females, rather than their having different liability thresholds.

Although multigenerational family studies of ASD are precluded by the low reproductive rate in people with ASD, studies of baby siblings of youth with ASD have been inconsistent in the extent to which the data support the expectations of sex differences in the multifactorial polygenic threshold models. Whereas Ritvo and colleagues^79^ and Sumi and colleagues^80^ showed increased risk of ASD in younger siblings of female probands with ASD, Ozonoff and colleagues^81^ found equal rates of ASD in siblings by sex of the proband. The higher threshold for females than males in the multifactorial threshold model of ASD has been attributed to the higher tolerance for mutational burden in females that has a protective influence on the development of ASD.^82^, ^83^ Evidence for this female‐protective effect in ASD has been showed in population‐based,^84^ family‐based,^85^, ^86^, ^87^ and cohort studies.^88^ The female‐protective effect has also been shown in ADHD,^89^ and may also apply to schizophrenia.^90^, ^91^ Genetic factors may also underlie sex differences in ADHD as showed by several family studies that show increased risk to the relatives of females compared with those of males with ADHD.^92^, ^93^, ^94^, ^95^

### 4.2 Twin studies

Twin studies allow researchers to disentangle genetic and environmental influences in psychiatric disorders. Because monozygotic (MZ or identical) twins share 100% of their genes, and dizygotic (DZ or fraternal) twins share, on average, 50% of their genes, comparisons between MZ and DZ twins are conducted to evaluate the degree of genetic and environmental influence on a specific trait and to calculate heritability estimates. Higher concordance between MZ twins than DZ twins, indicates a stronger genetic influence on a trait or disease, whereas similar concordance between MZ and DZ twins suggests greater environmental influences. Path analytic or variance component approaches that estimate the proportion of variance attributable to additive genes, common and unique environment have been the standard method of analysis of data from large twin studies. The combined twin family design is an even more powerful design that yields estimates of heritability and permits evaluation of multigenerational patterns of expression of genetic and environmental risk factors.^96^

Sex‐specific concordance rates can be used to infer differences in genetic and environmental factors underlying a trait or disease. Similar concordance rates among male‐male and female‐female pairs indicate similar underlying genetic factors, whereas differences may indicate either different genes or environmental exposures to the disease. Opposite‐sex twins are particularly informative in elucidating the relative contribution of genetic and environmental factors. if the same genes contribute to an outcome in males and females, then the opposite‐sex DZ twin pair correlation should lie between the correlations of the male‐male DZ and female‐female DZ pairs. However, if the correlation in opposite‐sex DZ twin pairs is significantly lower than that of the same‐sex pairs, the same genes do not contribute to the trait in males and females.^5^ Data from opposite sex twin pairs have been used as evidence for within utero “testosterone transfer”^33^, ^42^, ^97^ for some phenotypes such as epilepsy^98^ and language impairment.^99^ However, meta analyses of phenotypes such as body mass index and height have not supported the impact of prenatal hormone exposure on these phenotypes in later life.

There are numerous examples of twin studies that examined sex differences in the heritability of mental disorders including major depression, alcoholism, schizophrenia and ASD (eg, Taylor et al.^100^). Sex differences have been of particular interest for depression. In contrast to the lack of sex differences observed in familial transmission of depression, there is moderate evidence for greater heritability of major depression in women than in men. For example, in a sample of nearly 40 000 twins, Kendler and colleagues^101^ found a moderate correlation in genetic risk factors for depression in male and female twins, but that heritability was greater in women than in men (42% vs 29%), replicating his earlier evidence for a slightly greater estimate of heritability for depression in female than male twins.^101^, ^102^ Developmental twin studies have shown that the heritability of depression and anxiety emerges in early adolescence, with prepubertal depression more likely to reflect environmental influences.^103^

Sex differences in alcohol‐related outcomes have also been examined in numerous twin studies. For example, in the first population‐based twin study of alcoholism in the U.S., Prescott and colleagues^104^ found substantially higher concordance among MZ than DZ pairs across several definitions of alcohol abuse and dependence, there was no difference in the genetic liability between women (55‐66%) and men (51‐56%). A correlation of 0.50 was projected for same‐sex DZ twin pairs; whereas, they reported genetic correlations of 0.20 to 0.24 for opposite‐sex pairs, which indicates that there are different genetic influences for men and for women.^104^ In contrast to earlier work, a recent review concluded that although there are numerous sex differences for alcohol‐related outcomes, the genetic influence on these outcomes is the same across sexes.^5^ Future studies should aim to address these conflicting findings.

There are less consistent findings on sex differences in the heritability of ASD from twin research. Whereas early twin studies reported MZ twin concordance of 72%, compared with DZ twin concordance of 0%, with heritability estimate of 90%,^105^, ^106^ more recent work has shown lower MZ twin concordance rates (eg, 58% for male pairs and 60% in female pairs) and higher concordance rates for DZ pairs (eg, 21% for male DZ pairs and 27% for female DZ pairs). This narrowed distance in concordance rates between MZ and DZ twins yielded a substantially lower heritability estimate of 37% than that found in earlier studies.^107^ These studies did not show sex differences in the heritability of ASD. Sex differences may also differ across development. For example, because females have lower persistence rates of ASD,^11^ the sex ratio may differ in cross sectional studies as compared with studies of the trajectories of ASD across the life span. This may also apply to ADHD, because twin studies of ADHD have shown that the heritability of ADHD in youth is greater than that in adults.^108^ Prospective twin studies have shown that different genes may be associated with baseline symptoms compared with persistence of ADHD across development, but few of these studies have investigated interactions with sex.^109^ However, in contrast to family studies, twin studies of ADHD have found equal magnitude of heritability in males and females.

In summary, studies of familial transmission and twin concordance can be used to test hypotheses regarding differential genetic liability thresholds in males and females, different genes contributing to risk in males and females and differential expression of the disorders in males and females.

## 5 SEX DIFFERENCES IN MOLECULAR GENETIC STUDIES

### 5.1 GWAS/SNPs

With advances in the identification of polymorphic markers across the human genome, the case‐control study design has become increasingly popular in psychiatric genetics with sample sizes in some studies greater than 900 000 individuals. The Psychiatric Genomics Consortium (PGC, https://www.med.unc.edu/pgc/) has conducted genome‐wide meta‐ and mega‐analyses for multiple psychiatric disorders including ADHD, ASD, bipolar disorder, major depression and schizophrenia. Yet, sex differences in genetic architecture of these conditions has received relatively little attention. For example, the most recent GWAS that identified 102 variants associated with depression, controlled for sex as a potential confounding variable rather than examining its effect directly.^110^ The CONVERGE Consortium study of recurrent depression was restricted to female participants; however, the replication had participants of both sexes.^111^ Controlling for sex by including it as a covariate in GWAS is far more common than explicitly examining the effect of sex. In 2017, Powers and colleagues^112^ reported that only 1% of genetic association studies of any disease had reported sex differences, and an even smaller proportion considered sex chromosomes.

There is an emerging number of studies that have reported sex differences in the findings from GWAS of psychiatric disorders. Table 2 presents a summary of findings of recent GWAS that have presented sex stratified analyses, the number of genome‐wide sex‐specific significant SNP associations, and sex‐specific SNP based heritability (*h* ^2^_SNP_). There are significant sex differences for ADHD,^95^ alcohol dependence,^113^ ASD,^114^ anorexia,^115^ Major Depressive Disorder (MDD),^116^ Obsessive‐Compulsive Disorder (OCD),^117^ PTSD,^118^ and substance use disorder.^119^ Sex stratified GWAS have not been published for anxiety disorders, bipolar disorder or schizophrenia. However, there may be studies that have completed sex‐stratified analyses but did not report the findings if they were negative.^120^

**TABLE 2:** Sex differences in genome wide association studies

Disorder	Sex specific GWAS completed?	SNP associations (n: SNP or gene)		*h* ^2^_SNP_		Notes	Reference
		Male	Female	Male	Female		
Attention Deficit Hyperactivity Disorder	Yes	3	0	24.7%	12.3%	SNP IDs were not listed in the paper.	95
Alcohol Dependence	Yes	10: ADH1B, ADH1C, ADH4, GCKR, SIX3, SLC39A8, rs4936277, rs61902812, rs7906104, FTO	1: rs72716801	5.4%	11%	Predominantly male sample, so overall findings reflect the male signal.	113
Anxiety Disorders	No						
Autism Spectrum Disorder	Yes	2: rs7836146, rs7835763	3: rs60443693, rs12614637, rs140431641	Not reported	Not reported	The female‐specific dataset is underpowered for heritability calculation.	114
Bipolar Disorder	No						
Eating Disorders: Anorexia	Yes	Not reported	1: rs9812977	11%‐17%	11%‐17%	Top locus in combined sex analysis (8 hits) is the only hit in the female‐only analysis.	115
Eating Disorders: Bulimia	No GWAS						
Major Depressive Disorder	Yes	1: rs4478037	0	18%	22%		116
Obsessive‐Compulsive Disorder	Yes	0	2: GRID2, GPR135	13.1%	29.6%		117
Posttraumatic Stress Disorder	Yes	0	0	7%	29%		118
Schizophrenia	No						
Substance use (Opioid)	Yes	9 in ADGRV1	0	Not reported	Not reported	Lead SNP rs2030272 in the African American sample. No associations for European Ancestry.	119

Two approaches have been used to investigate sex differences in GWAS: (a) estimation of heritability using either genomic relatedness matrix restricted maximum likelihood (GREML), or linkage disequilibrium score regression, (LDSC) and (b) calculation of sex‐specific PRS, an average of risk alleles across the genome weighted by effect size and statistical significance.^121^ Hall and colleagues^116^ conducted sex stratified analyses in the UK Biobank and the Generation Scotland studies, and found similar SNP‐based heritability for depression in males (*h* ^2^_SNP_ = 0.18, SE = 0.06) and females (*h* ^2^_SNP_ = 0.22, SE = 0.06), whereas Duncan and colleagues^118^ report substantially higher SNP‐based heritability for PTSD in females (*h* ^2^_SNP_ = 0.21, SE = 0.09) than in males (*h* ^2^_SNP_ = 0.08, SE = 0.10). The depression study also describes the balance between modeling sources of heterogeneity including sex differences and disease subtypes in the overall sample, and the loss of power in stratified analyses that use only a portion of the sample. Likewise, Martin and colleagues^95^ hypothesized that under a liability threshold model, females with ADHD would have a higher genetic threshold (as represented by higher PRS) than do males with ADHD. However, no significant sex differences in the SNP‐based heritability of ADHD was found (female *h* ^2^_SNP_ = 0.123, SE = 0.025; male *h* ^2^_SNP_ = 0.247, SE = 0.021), nor was there an association between ADHD PRS with sex in cases, and the odds ratio for the PRS was the same in males and females. Consistent with expectations of the multifactorial polygenic threshold model they did find an increased risk of ADHD in relatives of females compared with males in a separate registry analysis.^95^ Reconciliation of discrepant findings from genomic and genetic epidemiologic analyses in these studies of depression and ADHD and other conditions will be an important future direction to gain understanding of the mechanisms underlying sex differences.

Khramtsova and colleagues^122^ eloquently reviewed both the methodological advances and challenges for studying sex as a biological variable in the molecular era. Recently, more molecular genetic research has been completed in a sex‐specific manner. For example, Randall and colleagues^123^ used sex‐specific GWAS to unravel the sexually dimorphic genetic underpinning of anthropometric traits, and found differential results for each trait by sex. It can be assumed that similar results will be found for other complex traits. Furthermore, Magi and colleagues^124^ reviewed the methodology for meta‐analysis of sex‐specific GWAS. They propose a sex‐differentiated test of association, which allows for heterogeneity of allelic effects between males and females. In their study, they completed simulations and report only a small loss in power for the sex‐differentiated meta‐analysis when the allelic effects of the causal variant are the same in males and females; however, when considering differing allelic effects between genders, their method offers substantial gains in power. Clearly, additional methodological investigation in this area is warranted. Finally, the Neale Lab (http://www.nealelab.is) at the Broad Institute has published publicly available GWAS summary statistics for the phenotypes in the UK Biobank (http://www.ukbiobank.ac.uk), for males and females combined and separately in an online repository (http://www.nealelab.is/uk-biobank) in order to allow researchers to examine sex‐specific associations. This resource should facilitate future comparisons of GWAS findings, SNP‐based heritability and PRS between males and females for a variety of psychiatrically relevant phenotypes.

### 5.2 Rare variants and CNVs

In recent years, more work has been completed to establish the role of rare genetic variants in neuropsychiatric disorders,^87^, ^125^, ^126^ including single nucleotide variants (SNVs) from whole genome or whole exome sequencing and CNVs, which can be either de novo or inherited mutations. Although these variants occur at low frequency, it is assumed that they would have a large effect.^127^ As described previously, there has been shown to be a “female‐protective effect” in ASD, which is supported by the higher incidence of these high impact rare variants among affected females than affected males.^128^

While we have concentrated on genotype variation in our discussion of molecular genetic studies of sex differences in psychiatric phenotypes, other “omics” domains may also play a role. Modifications, such as DNA methylation may have an impact on sex differences. In fact, Maschietto and colleagues^129^ postulated that a primary driver of sex differences in neuropsychiatric disorders is differential DNA methylation of autosomes by sex. The majority of the work in this area has been conducted in rodents, where it has been showed that DNA methylation plays a role in establishing sex differences in the brain during development, while profiles of epigenetic changes by sex during brain development in humans are not yet readily available.^130^ Differential gene expression patterns have been reported in male and female postmortem brain tissue, although it is unclear whether the expression and methylation differences result from, or are in the etiologic pathway of neuropsychiatric disorders.^131^ Recently, Xia and colleagues^132^ investigated the contribution of DNA methylation to sex differences in psychiatric disorders, and reported thousands of sex‐differentially methylated positions (DMPs) and regions (DMRs). Examining sex‐specific methylation and expression may be an important and underexplored avenue of research; the differential response by sex to environmental stress, as indexed by DNA methylation, may inform the differential expression of neuropsychiatric phenotypes by sex.

In summary, there is growing use of molecular genetic studies to compare the identity and strength of common SNP associations in males and females, the SNP‐based heritability of psychiatric disorders by sex, and the burden of both common polygenic risk and rare variants of large effect in affected males and females. Epigenetic and transcriptomic analyses have suggested differences in DNA methylation and gene expression in male and female brains, but the potential impact of these differences on risk and expression of psychiatric disorders remains an open question.

## 6 FUTURE DIRECTIONS

Advances in our understanding of sex differences in neuroscience provide new opportunities to study the role of genetic factors that may underlie mental disorders and their core components. For example, McCarthy and colleagues^32^ have provided a scholarly summary of our understanding of the role of sex hormones and immunological factors in the establishment of brain differences across development, and sex differences in regional brain volumes due to differential cell death, neuronal and glial genesis, dendritic branching and synaptic patterning between males and females. There are several strategies that may be employed to identify the role of genetic and environmental factors in the core domains underlying mental disorders. By integrating advances in neuroscience to study hypotheses for sex differences we can glean more information about how the sex differences in the brain lead to different sex ratios in complex disorders. Moreover, these studies may aid in identifying critical periods of risk, when exposure to environmental factors may influence genetic susceptibility factors, such as the prenatal period for neurodevelopmental disorders, middle childhood for ADHD, and adolescence and early adulthood for mood disorders. Studies of sex differences could also be more informative if they also considered sex differences in disease subtypes, age at onset, treatment response and other potential sources of heterogeneity.

Sex differences are also clearly important in pharmacologic treatment, yet females are not well represented in clinical trials. In fact, between 1997 and 2001, the majority of prescription drugs removed from the market (8/10) showed greater adverse effects in females.^133^ A salient example of this is a recommendation from the Food and Drug Administration (FDA) that the dosage of the sedative zolpidem be halved in women.^134^ This recommendation followed anecdotal evidence of impaired driving the morning after taking zolpidem among women.^135^ The ultimate decision was based on clinical trial data, as well as driving simulation studies.^136^ In this instance, it is assumed that women and men metabolize this compound differently, and this should have been established in earlier work.

To date, it is difficult to interpret the data regarding sex differences in the genetic architecture of psychiatric disorders, due to differences across studies in ascertainment and methods, which have yielded noncomparable samples in terms of severity, comorbidity and complications of mental disorders. The samples in current GWAS studies have not generally been systematically selected. Even though the large numbers may reduce the impact of some sampling biases, future studies should examine the effects of ascertainment, to examine whether findings may differ by the sex and severity of the disorder in the proband.

Family study data are central to establishment of the origin of mutations, that is, inherited or de novo, from both sequencing and structural variation data, and play a vital role in establishing the inheritance of both phenotypes and genotypes across generations.^137^ Evaluation of families will allow for the evaluation of both common and rare variants, as well allowing researchers to evaluate environmental risk factors in a way that is not possible in case‐control studies. Specifically including sex differences in the study design and prospectively studying sex differences across development in families will be vitally important to examining potential genetic and environmental mechanisms for sex differences. Systematic recruitment by sex of the proband and prospective designs that examine the sex‐ratio and potential influences across development may provide insight not only on the emergence of sex differences but also their underlying causes.

Another promising future direction is studies of sex differences in disorder endophenotypes that are posited to more closely reflect underlying genetic influences. There are several examples of studies that have identified endophenotypes for psychiatric disorders, including depression,^138^, ^139^ ADHD,^140^ ASD,^141^ and schizophrenia.^142^ Systematic exploration of sex differences in studies of endophenotypes could provide more comprehensive depiction of the spectrum of expression of genetic factors underlying major categorical diagnoses in psychiatry between the sexes. Similar to the large case‐control GWAS, most studies of endophenotypes (or subphenotypes) of schizophrenia and bipolar disorder do not systematically investigate sex differences.^143^

Prospective studies that systematically build sex into the design to study the longitudinal evolution of sex differences in males and females are needed. For example, in a prospective study of the course of young children at the initial diagnosis of ASD, Szatmari and colleagues^11^ showed that the sex of the affected child with ASD was the only significant predictor of differential trajectories of symptoms of ASD over time. They found that boys had more stable, severe symptoms over time, whereas girls exhibited less severe symptoms and improvement over time. In fact, some girls no longer manifest the cognitive and language problems at follow up. This illustrates that the age or developmental stage at ascertainment of a condition may influence the sex ratio. Developmental studies of sex differences across the life course will also aid in our understanding of the factors underlying these differences. Biologic and social factors in early life development, such as parenting style or social environment, have long been seen as clinically relevant to later adult psychopathology,^133^ but there remains a dearth of information on how these early factors may differentially impact the sexes.

Family history information is not typically collected in case‐control GWAS, because detailed family interviews of psychiatric disorders is generally beyond the scope of large sample size case‐control GWAS and health registry studies. However, enrichment of these large‐scale studies through systematic collection of family history information could inform our understanding of sex differences in the genetics of neuropsychiatric disorders, by enabling the examination of sex‐based rates and transmission in the family. Collection of family history in electronic health records (EHR) is typically limited to identification of affected cases from the clinical notes without denominators (ie, “Does anyone in your family have depression/alcohol abuse/etc.?” without enumerating family members) precluding estimation of recurrence risks.^144^ However, an exemplary study that mined emergency contact data from the next‐of‐kin contact information in the EHR to identify familial relationships with validation of the EHR next‐of‐kin relationships through genetically calculated kinships computed heritability estimates for a range of clinical conditions. They reported a median heritability of 0.41 (ICD‐9) and 0.31 (ICD‐10) for mental health disorders, but note in their discussion that mental health conditions are generally not well documented in EHRs.^145^ In fact, only moderate agreement has been reported for mental health diagnoses from administrative data, that is where an ICD, DSM or other similar reference standard diagnosis is compared with psychiatric diagnoses in routinely recorded data (median kappa = 0.45‐0.55).^146^ Large‐scale population registries will provide a valuable resource for identifying the role of sex differences in the prevalence, course and role of genetic and environmental risk factors for mental disorders. Moreover, systematic collection of additional family history information, including enumeration of family members, in large scale case‐control studies could inform our understanding of sex differences in the genetic architecture of neuropsychiatric disorders.

## 7 CONCLUSION

Sex differences of some kind—in prevalence, age at onset or presentation—have been demonstrated for a large portion of major psychiatric disorders. These may be due to some combination of artifact, differential susceptibility to environmental insults in males and females, effects of sex chromosome composition or differential nature and impact of genetic effects in males and females. Genetic epidemiologic studies have identified differences in heritabilities of several disorders between males and females and have provided support for a higher burden of genetic risk in the less affected sex in some cases. Molecular genetic studies have showed different SNPs associated with a disorder in males and females or differing strengths of SNP effects in males and females, and have also provided support for differing heritabilities or burden of risk alleles by sex. However, no consistent patterns have emerged across disorders and for the most part the mechanisms underlying sex differences in psychiatric disorders remain unexplained.

## Data Availability

Data sharing is not applicable to this article as no new data were created or analyzed in this study.
